# Antifragile Behavior Change Through Digital Health Behavior Change Interventions

**DOI:** 10.2196/32571

**Published:** 2022-06-03

**Authors:** Benjamin T Kaveladze, Sean D Young, Stephen M Schueller

**Affiliations:** 1 Department of Psychological Science University of California Irvine, CA United States; 2 Department of Emergency Medicine University of California Irvine, CA United States; 3 Department of Informatics University of California Irvine, CA United States

**Keywords:** digital health behavior change interventions, behavior change, digital health, self-management, antifragile

## Abstract

Digital health behavior change interventions (DHBCIs) offer users accessible support, yet their promise to improve health behaviors at scale has not been met. One reason for this unmet potential may be a failure to offer users support that is tailored to their personal characteristics and goals. We apply the concept of antifragility to propose how DHBCIs could be better designed to support diverse users’ behavior change journeys. We first define antifragility as a feature of an individual’s relationship to a particular challenge such that if one is antifragile to a challenge, one is well positioned to benefit from facing that challenge. Second, we introduce antifragile behavior change to describe behavior change processes that leverage person-specific antifragilities to maximize benefits and minimize risk in the behavior change process. While most existing behavior change models focus on improving one’s motivation and ability to face challenges, antifragile behavior change complements these models by helping to select challenges that are most likely to produce desired outcomes. Next, we propose three principles by which DHBCIs can help users to develop antifragile behavior change strategies: providing personalized guidance, embracing variance and exploration in choosing behaviors, and prioritizing user agency. Finally, we offer an example of how a DHBCI could be designed to support antifragile behavior change.

## The Need for More Effective Digital Health Behavior Change Interventions

Improving health behaviors such as eating, exercise, and sleep can profoundly impact one’s life. Nonetheless, behavior change is a notoriously difficult and deeply personal process. To support health behavior change, a variety of digital tools and services, called digital health behavior change interventions (DHBCIs), have been developed. Millions of people around the world already use DHBCIs via apps, websites, and wearables, so making these tools maximally beneficial and minimally risky for users is an important goal [1].

Despite enthusiasm about DHBCIs’ potential to improve people’s health at scale, randomized controlled trials of DHBCIs have not provided strong evidence of improvements in health behaviors or outcomes [2]. Furthermore, a meta-analysis found that physical activity–based DHBCIs were helpful for users with high socioeconomic status (SES) but not for users with low SES, suggesting that more attention is needed to ensure that DHBCIs can support diverse populations [3]. Finally, most users do not engage with DHBCIs sufficiently to achieve intended outcomes [4,5]. In short, DHBCIs are less effective, less engaging, and less accessible than hoped. However, researchers have argued that greater integration of the behavior change theory, aligning with the affordances and limitations of mobile platforms, could improve DHBCIs’ effectiveness [2,6,7].

One reason for DHBCIs’ unmet potential may be their inability to offer dynamic support that accounts for users’ personal strengths, weaknesses, and goals. We propose that future DHBCIs will be more successful if they can provide users with information and motivation that is contoured to their needs, helping them make the most of their opportunities to gain from while avoiding barriers and excessive risks. Here, we present the term “antifragile behavior change” to define behavior change processes that are made more efficient by leveraging user-specific factors. Antifragility is a concept that has been applied to economic and biological systems, but its application to behavior change can help identify new ways to tailor and personalize DHBCIs. As such, we put forward principles for designing DHBCIs to support antifragile behavior change and offer an example of a DHBCI that aligns with these principles.

## Antifragile Behavior Change

### Antifragility

“Antifragile” is a term coined by Nassim Nicholas Taleb to describe systems that gain from disorder, stressors, or uncertainty (broadly, challenges) [8]. With persistent exposure to a particular challenge, things that are fragile to that challenge break, those that are robust to it stay intact, and those that are antifragile to it get better. Antifragility is not a mindset or other kind of psychological process—it only describes one’s objective likelihood of gain and loss from facing a specific challenge. As a property of individuals, antifragility is a feature of the relation between a person and a particular challenge; all else equal, a student who grows and improves from criticism of their work (ie, is antifragile to the challenge of criticism) will tend to outperform a student who crumbles under (ie, is fragile to) criticism as well as a student who ignores (ie, is robust to) criticism. Nonetheless, a student who is antifragile to criticism may also be fragile to other challenges, such as focusing during long classes.

### Antifragility in Behavior Change

**Figure 1:**
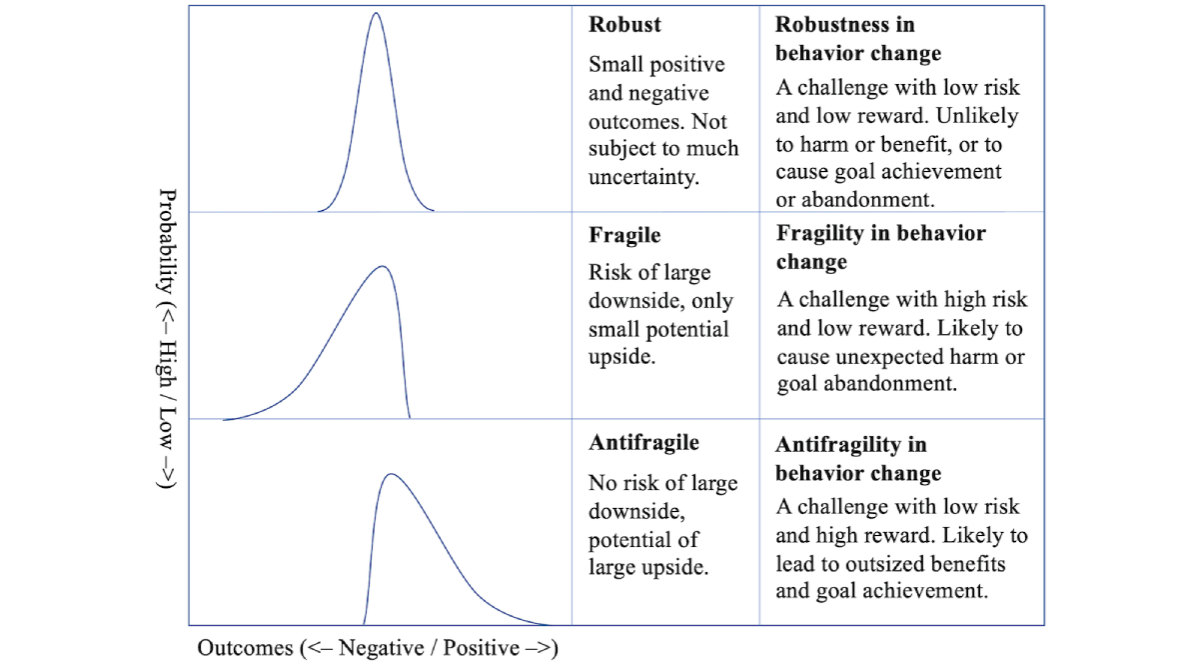
Mapping probability distributions to a behavior change context, positive (favorable) outcomes are viewed as progress toward successful behavior change or other benefits and negative (unfavorable) outcomes as movement toward giving up on one’s behavior change goal or other unintended consequences. In this model, each challenge has a unique probability distribution based on individual and contextual factors. As an individual continues exposure to a challenge, their outcomes can be seen as repeated samples from the challenge’s distribution.

Behavior change involves persistent and varied challenges, such as difficult workouts, diets, and competing time commitments. One’s level of antifragility, robustness, or fragility to a challenge determines the outcome of their exposure to it. If one is antifragile to a challenge, one is likely to derive outsized benefits from exposure to that challenge compared to their risk of harm. Inversely, if one is fragile to a challenge, exposure risks significant downsides and only affords minor possible benefits (Figure 1). Therefore, effectively choosing the challenges one faces is critical to successful behavior change: spending too much effort on challenges to which one is fragile is inefficient and potentially counterproductive, while not spending enough effort on challenges to which one is antifragile forgoes opportunity for gain.

Of course, it can be difficult to determine if one is fragile, robust, or antifragile to a given challenge; this uncertainty is inherent to the behavior change process. Exposure to a challenge can impact several domains of one’s life over varying timespans, so the benefits and risks of a challenge may be hard to predict and detect. Moreover, one’s relation to challenges is dynamic, changing as one progresses through the behavior change process and alongside various other life factors. Another source of this uncertainty is personal and situational context; one cannot be labeled as fragile or antifragile to a challenge without an understanding of the context in which one faces that challenge. For example, the potential outcomes of a 30-minute walk outside differ depending on factors such as one’s walking ability and their neighborhood’s safety. Addressing the meta-challenge of choosing which challenges to pursue, how much, and how often, is key for successful behavior change.

### Defining Antifragile Behavior Change

We define antifragile behavior change as a behavior change strategy wherein one faces challenges to which they are antifragile and avoids those to which they are fragile. We claim that such a strategy should be maximally efficient in aiding progress toward one’s behavior change goal while avoiding setbacks and other negative outcomes. Although the concept is intuitive (put most simply, “do what works for you, don’t do what doesn’t”), creating and maintaining an antifragile behavior change strategy requires detailed self-knowledge, careful monitoring of outcomes, and possibly expert guidance to help identify one’s opportunities, risks, and blind spots.

### Relation to Other Behavior Change Strategies

Antifragile behavior change provides a unique perspective that complements other models of health behavior change. While existing behavior change models generally focus on improving one’s motivation and ability to face challenges, antifragile behavior change asserts that these efforts are only useful if those challenges lead to desired outcomes. For example, models such as self-efficacy, mental contrasting, self-control, risk aversion, and a growth mindset are concerned with one’s beliefs and attitudes toward challenges [9-11]. Similarly, the Fogg Behavior Model (FBM) identifies how contextual factors might trigger, facilitate, or discourage facing a challenge [12]. Other models such as the START (Specificity, Timing, Acquisition, Rewards and feedback, and Tools) model aim to help individuals set actionable goals [13]. However, all of these models take for granted that the challenges they help one to pursue will efficiently lead to desired outcomes. Antifragile behavior change fills a gap in these models by providing a framework that informs which challenges to pursue and which ones to avoid.

This is not to say that attitudes and supports for behavior change are not important. First, these models are valuable within the context of antifragile behavior change because they shape the nature of a challenge, and, as such, the costs and benefits that the challenge carries. For example, approaching the challenge of swimming laps with a growth mindset, an attitude of playfulness, and a swimming buddy changes the experience and impact of that swim. Moreover, no matter how beneficial a challenge might be for someone, one must be willing to engage with it to reap those benefits (yet, it is also true that one can be antifragile to a challenge one finds odious and fragile to one they find irresistible). In sum, improving motivation and facilitating behaviors can help an individual effectively engage with challenges, which is necessary but not sufficient for improving outcomes [4].

### Antifragile Behavior Change in Practice

As an example of antifragile behavior change, a boxer training for a fight may choose to focus their efforts on sparring and meditation because they know that they are antifragile to those challenges, while also training in isolation with a strict diet because they know that they are fragile to the challenges of resisting distraction and temptation. Meanwhile, their extraverted opponent might choose to train around friends and family because they perform better with their support. Both boxers would almost certainly benefit from some training in all relevant domains, but the amount of time and effort that each boxer dedicates to each challenge should depend on their relations to those challenges.

Importantly, both boxers’ strategies would have been shaped by years of working with trainers to try out different challenges and find the ones that work for them. A trainer’s role is to identify their client’s fragilities and antifragilities, use that information to create a strategy that will maximize gains and minimize losses, and then provide structure and motivation to support the execution of the strategy. Next, we propose how a DHBCI can play the role of a trainer for users pursuing behavior change.

## Principles for Designing DHBCIs to Support Antifragile Behavior Change

### Overview

DHBCIs designed to support antifragile behavior change aim to make users’ behavior change journeys as productive as possible while avoiding serious downsides. We propose three principles for designing DHBCIs to support antifragile behavior change. First, to help users make the most of their antifragilities and avoid risk from their fragilities, DHBCIs should provide personalized guidance in the process of creating and implementing behavior change strategies. Second, to maximize opportunities to identify antifragilities, DHCBIs should encourage users to explore varied challenges. Third, to avoid unintended downsides, DHBCIs should prioritize user agency in decision-making by avoiding design choices that manipulate user choice and by explaining recommendations. We claim that these principles are flexible enough to be applied to a wide range of DHBCIs.

### Providing Personalized Guidance

DHBCIs designed to support antifragile behavior change should guide users through their unique behavior change journeys. This guidance should draw from evidence on effective behavior change approaches and be responsive to users’ ever-changing situations. To begin, DHBCIs should help users specify reasonable behavior change goals, drawing from evidence on effective goal setting and data on behavior change outcomes [14]. Next, they should assist users in selecting challenges to which they are likely to be antifragile and creating tailored behavior change plans based on those antifragilities. Finally, DHBCIs should support users in rigorously implementing their behavior change strategies, monitoring their progress, and making adjustments as necessary. This aligns closely with goal-oriented medical care, in which a patient and provider collaborate to provide care that matches a patient’s needs [15].

In each of these stages, DHBCIs can offer users support through information (eg, expert tips and suggested activities), structure (eg, setting a schedule and tracking their progress), and motivation (eg, personalized reminders and a compelling user experience) [16,17]. This guidance can be provided by human coaches, virtual conversational agents, or digital features such as text, images, or videos. Guidance should draw from scientific evidence on effective behavior change strategies and data on behavior change outcomes from similar others (if available) [16,17] and also adapt to personal factors.

### Embracing Variance to Identify Antifragile Opportunities

As noted, the meta-challenge of choosing which challenges to pursue and to what extent is central in antifragile behavior change. Addressing this meta-challenge requires an ongoing and dynamic information gathering process. To approximate one’s antifragilities, one can draw from evidence on behavior change and guidance from domain experts, but one should also personally engage in trial and error. This exploration is useful in the context of behavior change insofar as it can help one identify antifragilities (what Taleb calls “convex tinkering”) [8]. This process is also related to the exploration-exploitation dilemma, in which one must choose between a known and unknown outcome [18]. Once an antifragility is identified, individuals can shift to an exploitation strategy to maximize benefits in that area.

In light of this, DHBCIs should encourage users to continuously explore potential areas of antifragility, as long as such exploration is safe and potentially fruitful. Many current DHBCIs operate through repetition; for example, a DHBCI might send a user the same daily reminder or encourage a few features for repeat use. Instead, DHBCIs should promote exploration by leveraging variety in their design; this might involve offering a range of reminders, features, and interactions to help users find the approaches that work best for them. In addition, DHBCIs should support users in monitoring the impacts of their exploration by prompting user reflection and supporting data input. Importantly, exploration carries risks that should be weighed against its potential benefits; as we discuss below, a DHBCI should not introduce variation in a way that reduces user control.

### Prioritizing User Agency

Every behavior change journey involves a distinct set of challenges and facing any challenge may have a complex range of impacts on one’s life. Thus, decision-making in behavior change requires a holistic and detailed understanding of the person engaged in behavior change. Because one can likely predict and monitor outcomes from one’s own behavior change efforts better than an outside observer, one should have control over the decision-making process in their behavior change journey—this, again, aligns with goal-oriented medical care, which prioritizes patient decision-making over physician judgement [15]. This is true whether the external observer is a human or an automated system; while smartphone usage data can provide granular insights into a user’s behavior and mental state, it is far from providing a holistic understanding of a user’s life [19,20]. As such, we conclude that DHBCIs must be careful to provide support without undermining user agency.

Many DHBCIs employ nontransparent nudges, which attempt to relieve users of the psychological friction of making difficult choices by nudging them toward desired behaviors without their awareness [21]. In reducing users’ agency without sufficient information to reliably predict outcomes, these nontransparent nudges put users at risk of unintended downsides. The adverse spillover and second-order effects of these nudges into other domains of users’ lives are extremely difficult to predict and potentially costly [8,22]; a DHBCI designed to optimize one’s fitness will not simultaneously optimize one’s overall well-being or one’s roles as a friend and parent. Thus, efforts to shift user behavior without continuous user consent are inconsistent with antifragile behavior change.

In addition to avoiding nontransparent nudging, DHBCIs also need to encourage users to interact with their content critically. Specifically, DHBCIs should provide explanations to justify and contextualize their recommendations. For example, if a smartwatch running app suggests that a user take a rest day, it should explain why it is doing so and indicate its confidence in that suggestion (eg, “for most people with your level of experience, taking a rest day after training as hard as you did today is moderately helpful for building long-term endurance and avoiding injuries”). Without such explanations, a user might overestimate the accuracy or impact of a recommendation that is ultimately not well suited to their needs. In addition to prioritizing user agency in decision-making, explaining recommendations can increase DHBCIs’ effectiveness and trustworthiness [23].

As in the Greek myth of Procrustes, who, in an attempt to give each of his guests a perfect night’s sleep, stretches them out or amputates their legs to make them exactly fit his bed’s length, a DHBCI that attempts to optimize behavior change from the outside can misjudge users’ needs and cause unintended consequences [8]. As DHBCIs become more ubiquitous and engaging in the coming years, eliminating the risk of iatrogenic effects will be increasingly important. DHBCIs designed for antifragility should avoid using nontransparent nudges and offering recommendations without explaining them. Instead, they should prioritize user agency by providing transparent support that reflects the best available evidence, while also explaining the logic, strengths, and limitations of that support. Such guidance can empower users to make informed decisions within the dynamic contexts of their lives.

Based on the design principles we proposed—personalizing guidance, embracing variance, and prioritizing user agency—we offer a brief conceptualization of how well a DHBCI supports antifragile behavior change in Textbox 1.

**Considerations to determine how well a digital health behavior change intervention aligns with our principles of design to support antifragile behavior change.**

Does the DHBCI…

- Principle: personalize guidance
  Help users consider possible costs and benefits of challenges?
  Help users monitor the impacts of their choices over time?
  Allow users to personalize their behavior change strategies?
  Change based on new information or feedback from users?
- Principle: embrace variation and exploration
  Encourage users to try new challenges or interactions?
  Introduce a variety of features over time to support exploration?
  Prompt user reflection on potentially unhelpful patterns?
- Principle: prioritize user agency
  Avoid nudges that could influence user behavior without consent?
  Appropriately describe its limitations and uncertainty?
  Provide users clear explanations for its recommendations?

### Alignment With and Differences From Other Perspectives on DHBCIs

#### App Behavior Change Scale

Our design principles are consistent with some of the recommendations for DHBCI design outlined in the App Behavior Change Scale (ABACUS), a tool used to determine the behavior change potential of apps [24]. Specifically, our model aligns with the ABACUS’s recommendations that apps should allow users to “customize and personalize features,” provide “information about the consequences of continuing and discontinuing behavior,” help with “goal setting,” and offer “opportunity to plan for barriers” [24]. Antifragile behavior change explains that this personalization is valuable because the costs and benefits derived from challenges are person- and context-specific.

#### FBM

The FBM, which is based on the observation that motivation, ability, and a prompt are necessary for a desired behavior to take place, has been influential in behavior change and user experience design more generally [12]. Some interpretations and applications of the FBM attempt to support behavior change by promoting nontransparent nudging or other ways of influencing behavior without consent [21]. Our design principle of prioritizing user agency is opposed to such approaches. Nonetheless, the FBM also provides strategies for improving user motivation and simplifying tasks that could be useful for behavior change. We argue that integrating strategies from the FBM, such as increasing user ability to complete behaviors, considering timing and triggers, and addressing core motivators, can be helpful as long as those design choices are employed transparently and do not infringe on user agency [12].

#### Efficiency Model of Support

Our approach parallels Schueller, Tomasino, and Mohr’s Efficiency Model of Support [25], which considers ways to improve the efficiency of human support in behavioral intervention technologies (efficiency is defined as the ratio of benefit accrued to resources devoted to providing that support). Antifragile behavior change concerns the efficiency of the behavior change strategy as a whole: an efficient strategy maximizes progress toward one’s goal while minimizing the chance of abandoning the goal, as visualized in the antifragile distribution in Figure 1. In addition, consistent with our focus on person-specific strategies, the efficiency model of support emphasizes that the ideal type of support is contingent on person-level characteristics and goals [25].

## Example of a DHBCI Designed to Support Antifragile Behavior Change

Here we sketch an outline for a mobile app–based DHBCI designed to support antifragile behavior change for building physical strength. Our example draws from existing approaches to technology-supported behavior change, including behavior change techniques such as social support and instruction on performing behaviors [16,17,26]. It also aligns with the conceptual and technological architecture proposed by the Behavioral Intervention Technology Model [27]. Finally, it follows McCallum et al’s [28] “n-of-1 evaluation framework for behavior change applications” to inform which user variables are collected, aiming to gain information that can be aggregated and used to improve tailoring with high internal, external, and social validity [28]. Rather than introducing new features, our aim is to demonstrate how our design principles might be implemented in a DHBCI similar to existing ones by highlighting or modifying features with demonstrated real-world effectiveness.

**Figure 2:**
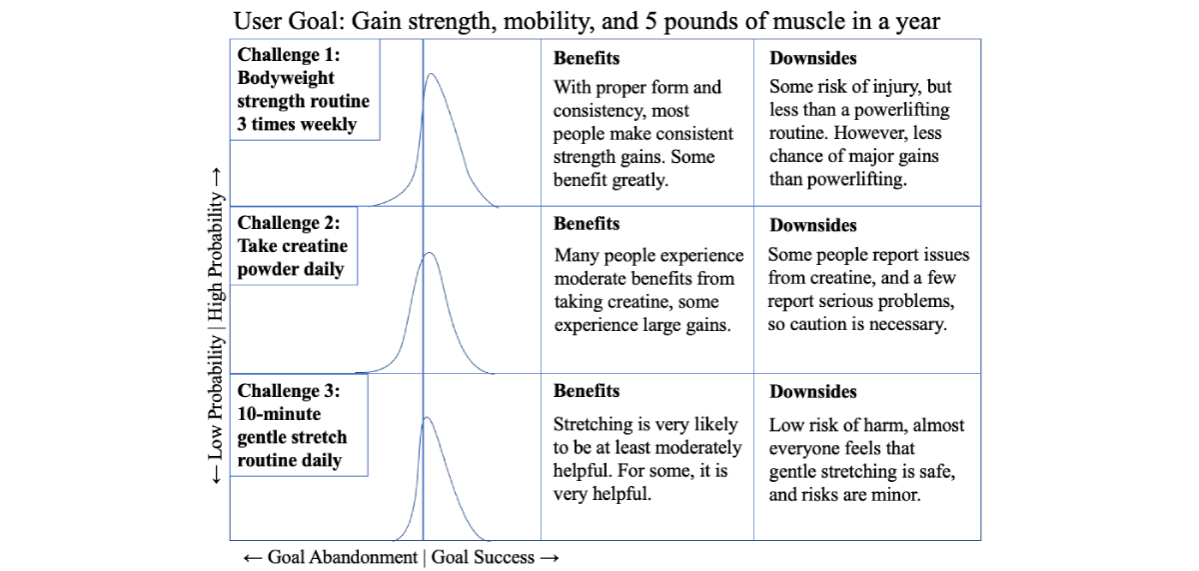
Three challenges a user might select to incorporate in their behavior change strategy during the planning phase of our digital health behavior change intervention example. The app recommends these challenges to a user based on challenge outcome data reported by other app users with similar characteristics and goals. The initial probability distributions shown are based on outcome data from other users, but as a user reports outcomes from their efforts, their predicted outcome distribution for each challenge is updated.

The app begins with a planning phase, in which the user generates a behavior change strategy, followed by an execution or evaluation phase, in which the user provides data to monitor their progress and adjusts their strategy if needed. The app’s user interface centers on visualizations of predicted outcomes of each challenge, in which “prior” predicted outcomes on the basis of information crowdsourced from other users are updated as a user reports their experiences, forming increasingly personalized “posterior” probability distributions (Figure 2).

To begin the planning phase, the app provides the following brief introduction:

In the words of C.T. Vivian, ‘You are made by the struggles you choose.’ To change your behavior, you have to put in effort to face challenges, such as intense workouts or maintaining a good sleep schedule. Choosing the right challenges for you will bring you closer to your goal, but facing the wrong challenges could hold you back or even cause injury. This app will help you to find and maintain the right behavior change strategy for you, but remember that you know yourself best, so you should always consider the app’s recommendations in the context of what is right for you and those around you.

Note that although this introduction explicitly discusses concepts relevant to antifragile behavior change, a DHBCI can be consistent with our design principles without doing so.

Continuing the planning phase, the app asks the user for some personal context, such as their behavior change goal, goal timeline, age, and experience with physical exercise. Based on this information and data from similar people who used the app previously, it presents a series of suggested behavior change challenges that the user can select from to create their strategy. These challenges might include a regular weight lifting regimen, a stretching routine, a macronutrient goal, and refraining from drinking alcohol. Figure 2 provides an example of how these challenges might be presented to users, including distributions to help users visualize risks and opportunities from each challenge. Further, the app provides a series of support options for the user to choose from; for example, motivational nudges and personalized recommendations from the app, instruction on challenges, or access to a peer support community to exchange advice and encouragement. These support options can be further customized, allowing the user to choose the app’s tone (eg, kind and funny or tough and demanding) or their support community’s makeup (eg, women from the age group of 18-30 years or people interested in bodybuilding). Finally, the app creates an editable schedule with all of the user’s selected challenges.

During the execution or evaluation phase, the user completes challenges when scheduled and reports positive and negative impacts that they believe to have resulted from each challenge. These data are used to generate personalized suggestions for adjusting one’s behavior change strategy and to update their predicted outcomes. For example, if a user consistently experiences more downsides than upsides after weight training, the app might recommend power yoga to achieve the same goal from a different approach. Throughout this phase, the app also encourages the user to continue exploring new challenges to discover new areas of antifragility. Finally, the user’s outcome data are aggregated with other users’ data to contribute to the app’s knowledge base of behavior change strategies. Because it gains aggregate information from many users’ successes and failures, the app itself can be seen as antifragile to its users’ efforts.

Figure 2 shows the three challenges a user might select to incorporate in their behavior change strategy during the planning phase of our DHBCI example. The app recommends these challenges to a user on the basis of challenge outcome data reported by other app users with similar characteristics and goals. The initial probability distributions shown are based on outcome data from other users, but as a user reports outcomes from their efforts, their predicted outcome distribution for each challenge is updated.

## Future Directions

Our paper presents antifragile behavior change as a theoretical concept that can be useful for DHBCI design. Future work can test this concept and potentially validate and extend it. First, advancing the understanding of antifragile behavior change will require measures to assess whether DHBCIs conform to the principles we proposed. Textbox 1 provides some conceptual considerations of how well a digital intervention aligns with antifragile behavior change, which might be adapted into a formal measure or assessment. Once a measure is created and validated, it opens up the possibility of addressing various research questions; for example, how well do different commercially available DHBCIs use antifragile behavior change principles, and do such interventions result in greater impact or engagement? Second, both researchers and developers could integrate antifragile behavior change principles into their designs. For researchers, this would enable tests of antifragile behavior change’s effectiveness: comparing DHBCIs and DHBCI features that align with the principles of antifragile behavior change to those that do not in terms of behavior change, health, and engagement outcomes. For developers, this represents another way to incorporate conceptually grounded behavior change techniques into their products. Third, research is needed to test our design principles across different DHBCIs and populations, examining the externalities of antifragile behavior change and trade-offs between strategies. For example, removing design elements that could threaten user agency might make it harder to tailor content, thereby decreasing some users’ willingness to engage with an app’s recommendations. These directions could help extend our suggestions into research and applications for the field.

## Conclusions

DHBCIs hold great potential to help people improve their health-related behaviors and overall well-being, but their promise has been held back by a failure to provide support that fits the contours of diverse users’ lives. Viewing DHBCIs through the lens of antifragility reveals both opportunities for advancement and reasons for caution. We presented three principles for designing DHBCIs to support antifragile behavior change. First, DHBCIs should provide personalized guidance to fit user-specific fragilities and antifragilities throughout the behavior change process. Second, DHBCIs should encourage users to explore varied challenges to discover new areas of antifragility. Third, DHBCIs should not limit user control in an attempt to make behavior change easier, as such efforts to optimize users’ lives from the top down can inadvertently cause harm. We claim that integrating these principles into DHBCI design will improve these tools’ abilities to support users across diverse populations. We hope that this paper will spark interest in reconsidering DHBCIs from the perspective of antifragile behavior change, both among DHBCI developers and behavior change researchers.

## Abbreviations

ABACUS: App Behavior Change Scale
DHBCI: digital health behavior change intervention
FBM: Fogg Behavior Model
SES: socioeconomic status
START: Specificity, Timing, Acquisition, Rewards and feedback, and Tools
